# Editorial: Neurobiological Models of Psychotherapy

**DOI:** 10.3389/fnbeh.2019.00144

**Published:** 2019-06-26

**Authors:** Arash Javanbakht, Cristina Maria Alberini

**Affiliations:** ^1^Stress, Trauma, and Anxiety Research Clinic (STARC), Department of Psychiatry and Behavioral Neurosciences, Wayne State University, Detroit, MI, United States; ^2^Center for Neural Science, New York University, New York, NY, United States

**Keywords:** psychotherapy, neuroscience, neurobiology, psychoanalysis, cognitive therapy, behavioral therapy, anxiety, trauma

The last decade has witnessed an exponentially growing interest in integrating neuroscience into psychotherapy. While neuroscience addresses the mechanistic understanding of brain functions by framing specific questions, psychotherapy examines the richness of complex clinical and individual behavior and history. Understanding the biological bases of complex behavior, human brain-mind functions, as well as their maladaptive responses, and identifying scientific approaches to assess how psychotherapy can help psychopathologies would significantly transform the approaches to mental health and diseases.

Psychotherapy is an individualized yet comprehensive biological treatment; it does not target one receptor, one or two neurotransmitters, or single modulators; it taps into all the biological regulations underlying complex brain responses. The end result of this type of intervention is a re-elaboration of the whole sense of self and others, through new learning and new experiences that encompass cognitive, emotional, and internal regulation processes. Successful therapies produce comprehensive, lasting, measurable physical changes in the brain.

In the past few decades, the progress in neuroscience research has provided a much deeper understanding of the brain structures and functions; applying this understanding and neuroscientific methodology to psychopathologies and therapeutic interventions can be transformative for advancing mental health. Neuroscience research is now, in fact, able to identify the genetic, epigenetic, anatomical, circuitry, and functional bases of behavioral manifestations. Studies in non-human animal models have provided important knowledge for testing hypotheses in humans in both healthy conditions and diseases and have unraveled a number of mysteries of many diseases. Psychotherapy, on the other hand, offers years of clinical experience and a rich understanding of human behavior, but still lacks empirical assessments and methodologies. Therefore, integrating knowledge and methods of neuroscience and psychotherapy will exponentially advance the formulation of new hypotheses, and therefore the comprehension and treatments of mental states and diseases. Given the complexity and variety of human mental functions and diseases, both disciplines, but especially their integration, are still in their infancy, and will require a great amount of work and investment in order to advance relatively rapidly.

One major question that can be readily investigated is whether, how and what types of changes are produced by psychotherapy. The answer to this question will inspire and promote the development of more effective, long-lasting, and integrated therapeutic methods.

This Research Topic “*Neurobiological Models of Psychotherapy*” brings together basic, clinical, and translational neuroscience research with psychotherapy theories, knowledge and clinical approaches to discuss evidence that psychotherapy changes the brain. The discussions in this research topic suggest new integrated knowledge to understand mental health and treat diseases.

Firstly, Solms (University of Cape Town, Cape Town, South Africa), discusses the neurobiological underpinning of the psychoanalytic theory, particularly focusing on the claims concerning innate emotional needs, learning from experience, and unconscious mental processing. On the basis of these claims, he also presents the neurobiological underpinnings of the mechanisms of psychoanalytic treatment, and, finally, he provides a review of the available empirical evidence of psychoanalytic therapeutic efficacy. Cabaniss (Columbia University, New York, NY, USA) underlines the importance and impact of teaching neuroscience to psychotherapy trainees and presents the crucial contributions of five papers that she uses in her teaching of psychotherapy. Radulovic et al. (Northwestern University and the University of Chicago, Chicago, IL, USA) discuss the importance of clinical, cognitive, and neurobiological perspectives on memory research relevant to dissociative amnesia. Zilcha-Mano et al. (University of Haifa, Israel, and Columbia University, New York, USA) review the literature regarding the neurobiological underpinnings of therapeutic alliance and expectancy and emphasize the importance of neurobiological studies to understand these effects. Scult et al. (Duke, Cornell, Kent State, Case Western, Arizona, CUNY, Columbia Universities, USA) report evidence that Emotion Regulation Therapy (ERT) change brain resting-state functional connectivity. Brockman (Columbia University, New York, NY, USA) describes his personal experience as an example to critically discuss what he believes psychoanalysis is lacking, and suggests ideas about how psychoanalysis needs to be integrated with the evidence-based neuroscientific approach.

Two articles discuss behavioral therapies of posttraumatic stress disorder (PTSD): Stojek et al. (Emory University and VA, Atlanta, GA, USA) present the current knowledge on how prolonged exposure therapy impacts the neural circuits related to PTSD, and discuss neurobiological enhancements that have been or may be used in conjunction with prolonged exposure therapy to enhance its effectiveness. Watkins et al. (Emory University, Atlanta, GA, USA) review and discuss the methodological guidelines indicated by the Veterans Health Administration and Department of Defense (VA/DoD) and the American Psychological Association (APA) in 2017 for PTSD treatment.

Finally, pointing at the robust overlaps of the phenomenology, neurobiology, and therapies of anxiety and trauma related disorders, Javanbakht (Wayne State University, Detroit, MI, USA) proposes potential overlapping neurobiology of seemingly different therapies of these disorders including psychoanalysis, cognitive, and behavioral therapies.

## Author Contributions

All authors listed have made a substantial, direct and intellectual contribution to the work, and approved it for publication.

### Conflict of Interest Statement

The authors declare that the research was conducted in the absence of any commercial or financial relationships that could be construed as a potential conflict of interest.

